# Time, population mobility, and HIV transmission

**DOI:** 10.1016/S2352-3018(19)30413-8

**Published:** 2020-01-14

**Authors:** Susan Cassels

**Affiliations:** a Department of Geography, University of California Santa Barbara, Santa Barbara, CA 93106-4060, USA

Population mobility is an intrinsic human condition. People have always moved over space and time, and always will. Yet mobility can be hard to define and measure, and thus it is challenging to understand the effects of mobility on the spread of HIV. In *The Lancet HIV*, Oliver Ratmann and colleagues^1^ combined social and demographic data with phylogenetic data in an innovative way to measure whether migration led to the spread of HIV from high HIV prevalence fishing communities along Lake Victoria to neighbouring inland communities in south-central Uganda. They asked this important question because HIV prevention efforts and treatment interventions are often prioritised to geographical hotspots, with the implicit assumption that these interventions will also have indirect benefits in neighbouring areas due to infections disseminating from the hotspot. They did not find frequent cross-community HIV transmission between Lake Victoria hotspots and nearby inland populations based on 293 source–recipient pairs with support for epidemiological linkage and direction of transmission. They concluded that, in this particular setting, targeted control of HIV in the lakeside hotspots would have a minimal effect on the epidemic elsewhere.

As countries move toward the UNAIDS 90-90-90 treatment goals and beyond, we will need more studies like that of Ratmann and colleagues to identify and predict whether and how population mobility will influence (positively or negatively) HIV intervention effectiveness. Strategies to end the HIV epidemic, especially those that target geographical hotspots, will increasingly need to account for population mobility with special attention to time.

Human mobility directly and indirectly affects HIV transmission dynamics over time, but the temporal scale has received less attention. For example, the importance of population mobility on intervention effectiveness in a community depends on the relative timing of the intervention and mobility in the population.^2^ A study by the same research group in Rakai, Uganda, found that HIV incidence among recent migrants remained much higher compared with non-migrants despite a scale up of combination HIV prevention efforts.^3^ One posited explanation is that prevention services did not effectively reach recent migrants, as also seen in the ANSR 12249 Antiretroviral Treatment as Prevention trial in South Africa.^4^ Relative timing of migration with HIV-related risk behaviours and barriers to engagement in care could lead to disproportionate ongoing transmission. Additionally, the timing of mobility over the life course is important to consider, as the risk of HIV acquisition or transmission also varies with age and events like marriage and pregnancy. In a study from Malawi, individuals infected with HIV were more likely to migrate, often after marital dissolution.^5^ Another study from 2018 examined women's migration in the context of pregnancy and post partum, and found frequent mobility and complex challenges to retention in post-partum HIV care.^6^

Migrant populations are heterogeneous, and the relative importance of different types of mobile populations on HIV depends on the stage of the epidemic. At the start of the HIV epidemic in sub-Saharan Africa, the spread of HIV was closely tied to migrant flows,^7^ such as labour migration. Nowadays, mobility in sub-Saharan Africa is complex, comprising circular migration, rural–urban migration, and counter-urbanisation. Generalised mobile populations tend to be at higher risk of HIV acquisition and onward transmission,^3^, ^8^ and the relationship is highly gendered.^9^ As we reach the end of the epidemic, we should prioritise HIV interventions that directly address mobility among the most vulnerable populations, such as ethnic and sexual minorities and poor populations. The remaining 10-10-10 populations will be disproportionately mobile populations who face legal challenges, stigma, discrimination in accessing HIV services, and housing insecurity.

Migration can have non-linear and long-lasting effects on transmission dynamics, depending on the epidemic's stage, the frequency and context of migration, the relative timing of interventions, life-stage, and the sexual behaviour of migrants.^10^ Prospective study designs or mathematical models of HIV transmission dynamics are needed to understand the broad effects of population mobility on HIV transmission over time.
